# Development and psychometric evaluation of a quality of life questionnaire for infertile women: a mixed method study

**DOI:** 10.1186/s12978-020-00988-7

**Published:** 2020-09-10

**Authors:** Zahra Kiani, Masoumeh Simbar, Sepideh Hajian, Farid Zayeri

**Affiliations:** 1 Student Research Committee, Department of Midwifery and Reproductive Health, School of Nursing and Midwifery, Shahid Beheshti University of Medical Sciences, Tehran, Iran; 2 Midwifery and Reproductive Health Research Center, Department of Midwifery and Reproductive Health, School of Nursing and Midwifery, Shahid Beheshti University of Medical Sciences, Tehran, Iran; 3 Department of Midwifery and Reproductive Health, School of Nursing and Midwifery, Shahid Beheshti University of Medical Sciences, Tehran, Iran; 4 Proteomics Research Center and Department of Biostatistics, Faculty of Allied Medical Sciences, Shahid Beheshti University of Medical Sciences, Tehran, Iran

**Keywords:** Study protocol, Sequential exploratory mixed-method study, Validity, Reliability, Psychometric evaluation, Infertility, The quality of life questionnaire for infertile women, QOL-QIW

## Abstract

**Background:**

Infertility is one of the most important issues that negatively influences women’s quality of life, especially when the cause is associated with females. Given that no instruments have been designed to assess quality of life among infertile women with focus on female factors, this study was conducted to develop and evaluate the psychometric properties of a female-centric quality of life questionnaire for infertile women.

**Method:**

This sequential exploratory study was conducted in two stages. First, the concept of quality of life and its dimensions as they relate to infertile women were elucidated through a qualitative inquiry accompanied with a content analysis. Accordingly, infertile women and key informants from a teaching hospital affiliated with the Mazandaran University of Medical Sciences and a private center for infertility treatment in Sari (north of Iran) were screened through purposive sampling until data saturation. Those who satisfied the inclusion criteria and exhibited maximum variance in terms of age, educational level, employment status, infertility duration, treatment type, and social class were recruited. The conventional content analysis was carried out in accordance with the steps proposed by Graneheim and Lundman, and the accuracy and robustness of the data were verified using Lincoln and Guba’s criteria (credibility, transferability, dependability, confirmability and authenticity). Second, the psychometric properties of the instrument developed in the qualitative stage were evaluated using a quantitative method and on the basis of the results of a literature review. The content, face, and construct validity of the instrument was determined, and its test–retest reliability and stability were ascertained using internal correlation and Cronbach’s alpha. The collected data were entered into the Statistical Package for the Social Sciences (version 22) for analysis, and descriptive statistics were calculated.

**Discussion:**

Developing and evaluating the psychometric properties of a valid and reliable female factor-centric instrument that measures quality of life among infertile women will be very useful in the assessment of their future status.

## Plain English summary

Infertility is a crucial issue that is affected by different cultural and social factors. In many societies, infertility is seen as a female problem, and the failure of women to achieve pregnancy is regarded as a major shortcoming. These orientations impose considerable family and social pressures on women. Between couples, women are the most strongly affected by infertility because these individuals may suffer the consequences of a divorce and grapple with loneliness, in addition to being subjected to stigmatization from society. Most women also have no income, particularly in developing countries, so treatment costs are paid for by their husbands. These situations expose women to tremendous stress, which in turn, affect their quality of life. Considering this adverse effect, developing a valid and reliable instrument for assessing the quality of life of this population is necessary. Results can be used as reference in targeting, prioritizing, and allocating resources as well as material and human facilities to satisfy the needs of infertile women. The instrument can also be used in studies that examine therapeutic, psychological, and social outcomes before and after intervention. With consideration for these issues, the current work was carried out in accordance with a qualitative and quantitative design. In the qualitative stage, the concept of quality of life among infertile women was elucidated through interviews with female respondents and key informants as regards female-related factors. Then, an appropriate instrument was developed on the basis of the qualitative findings and a literature review. The instrument’s psychometric properties were then quantitatively evaluated to determine its validity and reliability.

## Background

Infertility refers to the failure to achieve a clinical pregnancy after approximately 1 year of regular unprotected sexual intercourse [1]. As a global problem, it affects about 13% of women and 10% of men, with the causes of female infertility including problems in ovulation, fallopian tube issues, pelvic adhesions, endometriosis, and unexplained infertility [2]. A systematic study on the prevalence of infertility in 190 countries found that in 2010, the prevalence levels of primary and secondary infertility were 1.9 and 0.5%, respectively, in women aged 20 to 44 [3]. The World Health Organization (WHO) identified infertility as an important reproductive health problem [4] which differs from other disorders in that it does not threaten life but exerts devastating effects on individuals, families, and societies [5]. Unfortunately, this condition is accorded minimal attention [6], and most often, afflicted individuals struggle in silence—a situation that negatively influences their quality of life [7].

The deterioration of quality of life among infertile women is prompted by numerous factors. The inability to conceive can impose considerable pressure on women and lead to feelings of shame and guilt [8]. Women are also more susceptible to depression and anxiety than are men [9], and grappling with infertility reduces sexual attraction and sexual desire among the former [10]. These problems, at the individual level, can lead to psychological imbalance, separation between couples, and, ultimately, divorce [11]. At the social level, many infertile women suffer from social isolation and despair, with their performance affected by society, family, and culture. In developing societies, a woman is considered complete only when she becomes a mother, which leads to inequality between men and women and gender-driven suffering [12]. In many developing countries, as well, parenting is culturally mandatory. In these civilizations, infertility is traditionally regarded as a female problem, with blame placed on women, thereby bringing about tremendous social suffering for them [13].

Quality of life has been defined in various ways, and the WHO describes it as “individuals’ perception of their position in life in the context of the culture and value systems in which they live and in relation to their goals, expectations, standards and concerns.” It is a very broad concept that encompasses physical and mental health, level of independence, social relationships, personal beliefs, and an individual’s relationship with salient features of his/her environment [14]. Infertile women’s quality of life is an important issue that has recently been examined by health researchers. This aspect of living is important at the individual and social health levels because of its critical role in later periods of life [15]. Given this significance, accurately measuring quality of life is a crucial requirement that has motivated the development, use, and evaluation of a variety of instruments in different studies. In the majority of research, the infertile couples’ quality of life questionnaires developed by Yaghmaei et al. (2009) and Boivin et al. (2011) have been exclusively used. The findings of these studies indicated a lower quality of life in infertile women than in infertile men [16–19]. Conversely, general instruments designed to gauge quality of life (e.g., SF36 [36-Item Short Form Health Survey], WHOQOL [WHO Quality of Life Questionnaire] are designed to have a wide range of content and cover the most common areas of quality of life in the majority of society. The WHOQOL Questionnaire is designed with four dimensions, namely, physical health, mental health, social relations, and environmental health [20], while the SF36 concentrates on eight dimensions, namely, general health, physical performance, role restrictions owing to physical causes, role restrictions due to emotional causes, physical pain, social performance, energy and vitality, and mental health [21]. The main drawbacks of these instruments are that they disregard sexual, economic, and social dimensions, which are important to certain groups such as infertile women, and that the other dimensions considered in the questionnaires are insufficiently sensitive to measure changes in the quality of life of individuals suffering from different diseases [22]. Questionnaires that are specific to the quality of life of infertile couples [23, 24] are not free from deficiencies as these instruments are not geared toward examining the status of infertile women, despite the conventional perception of childbearing and parenting as the most prominent female roles and infertility as a female issue. Moreover, women are more vulnerable to the condition, and their suffering is more severe and profound than that of men [25]. The social structures that bring forth this agony, along with its consequences on the quality of life of women, differ from those that give rise to suffering among infertile men [25].

All the questionnaires designed to ascertain quality of life in relation to infertility are based on couples, and none are oriented toward gender. These questionnaires also pay no attention to infertility despite its definite influence on quality of life. One of the essential issues explored in the present research is that general and specific instruments for assessing quality of life among infertile couples are mostly outdated and do not use new methods of instrumentation (e.g., a content validity index) to verify the probability of chance agreement, degree of ease, response rate, ceiling and floor effects, standard error of measurement, and minimal detectable change. These factors are crucial to the development and psychometric testing of quality of life scales. The lifestyles of infertile women have changed over time, and the various problems that they encounter at the individual, family, psychological, and social levels have given rise to different concepts associated with their quality of life. However, no instrument that can be used to measure this aspect with concentration on female factors, describe the status quo, and ultimately determine the effectiveness of interventions has been developed. The current study thus sought to use new knowledge in the field to create a valid and reliable instrument. The concept of quality of life as it relates to infertile women was first elucidated on the basis of female experiences and perceptions, after which an instrument was developed and evaluated in terms of psychometric properties.

### Objectives

This sequential exploratory research pursued the following objectives in the qualitative and quantitative stages of the research:

Qualitative stage:

- To explain the concept and dimensions of infertile women’s quality of life

Quantitative stage:

- To develop a comprehensive item pool for what this research calls the quality of life questionnaire for infertile women (QOL-QIW)
- To determine the qualitative and quantitative content validity of the QOL-QIW
- To ascertain the qualitative and quantitative face validity of the QOL-QIW
- To gauge the construct validity of the QOL-QIW
- To determine the response rate, degree of ease, and ceiling and floor effects of the QOL-QIW
- To measure the internal consistency of the QOL-QIW
- To determine the stability of the QOL-QIW

## Methods

**Fig. 1:**
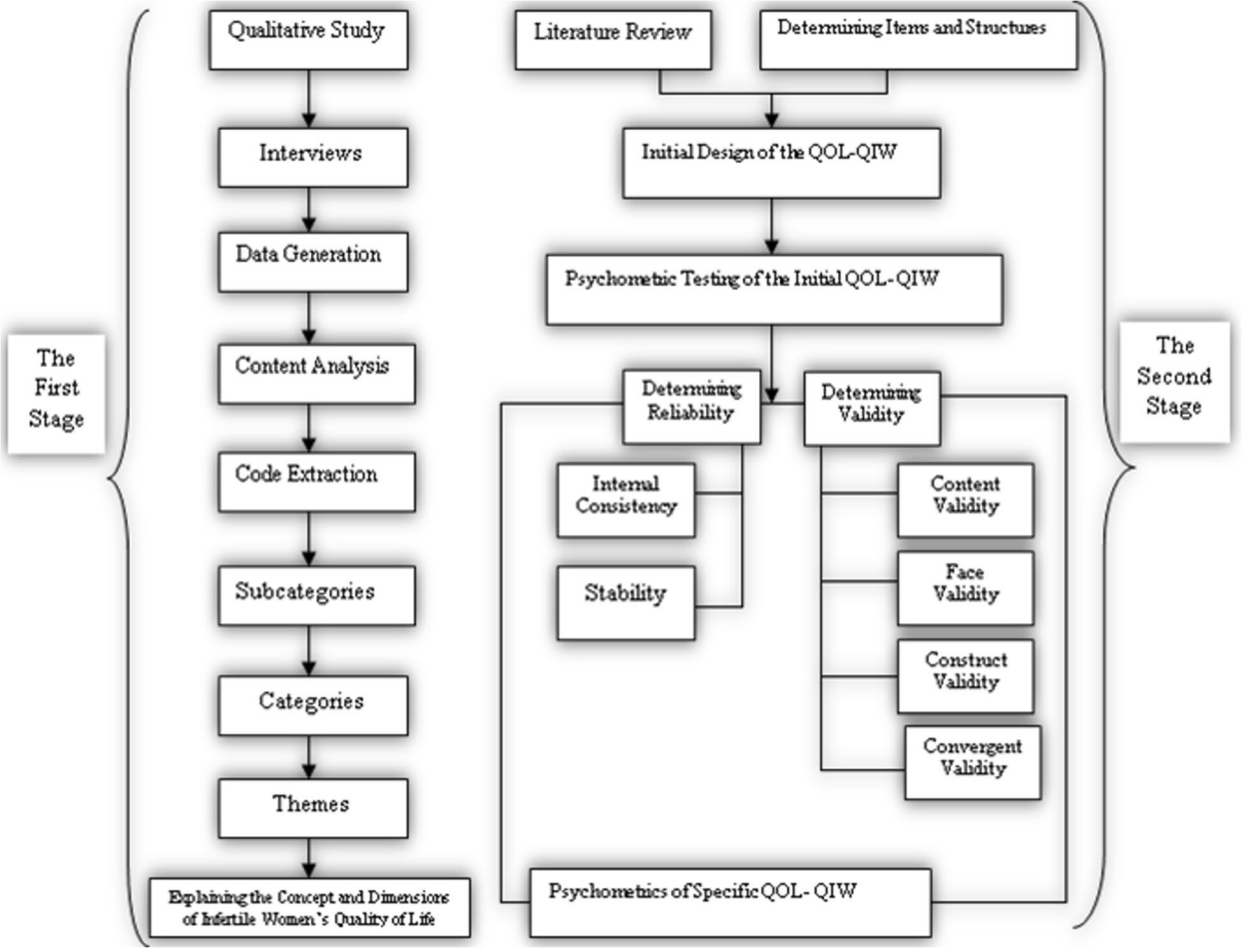
The sequential exploratory research process

Given the sequential exploratory nature of the study, it was initiated with a qualitative approach. Given the lack of quality of life examinations that underscore the female factors of infertility, a conventional content analysis was performed to explain this aspect of life from the perspectives of the respondents. Insights from these perspectives were drawn via an inductive–deductive approach. The questionnaire developed and evaluated in the qualitative stage was used as the data collection tool in the quantitative stage. The steps implemented in the research are illustrated in Fig. 1.

### Qualitative stage

#### Data collection

Data were collected via in-depth individual interviews, with pre-determined question guides as reference. The general question“ *how did you feel when you realized you were infertile?*"was employed as the overall interview guide.

#### Participant characteristics

The participants of the study were infertile women with the following characteristics:

1. Experiencing 1 year of infertility, with the diagnosis related to a female factor by a specialist
2. No infertility in their spouses
3. Having a formal marriage and living with their spouses
4. No adopted children
5. Willingness to participate in the study
6. Absence of psychological disease

The key informants had the following attributes:

1. Spouses, friends, or relatives of infertile women
2. Willingness to participate in the study

The key informants who provided services had the following characteristics:

1. Having at least a bachelor’s degree and 2 years of continuous work experience in an infertility treatment center
2. Care Provider (e.g., gynecologist, midwife, psychologist, reproductive health doctor, or sociologist)
3. Willingness to participate in the study

#### Research setting

The research was carried out in a site where the participants were living and their experiences took place or in locations selected by the participants [26]. The interviews with the infertile women were held in a quiet place in a teaching hospital affiliated with the Mazandaran University of Medical Sciences and a private infertility treatment center in Sari (north of Iran) or wherever the participants wished to hold the sessions.

### Sampling method

The participants were recruited via purposive sampling with consideration for maximum variance in terms of age, education, employment status, social and economic status, type of infertility, infertility treatment, and duration of infertility. Sampling was performed until data saturation.

### Data analysis

The data were scrutinized using the conventional content analysis steps proposed by Graneheim and Lundman, and the accuracy, validity, and robustness of the data were verified and enhanced using Lincoln and Guba’s (1994) criteria, namely, credibility, transferability, dependability, confirmability and authenticity.

### Quantitative stage

The questionnaire developed and evaluated after the qualitative phase was used to collect data during the quantitative phase.

### Content validity

The researcher asked 10 specialists (infertility subspecialists, reproductive health and social medicine specialists, psychiatrists, and health education specialists familiar with qualitative research) to provide feedback on the questionnaire as regards grammaticality, appropriate word use, and appropriate placement of phrases. The specialists were also instructed to qualitatively assess the adequacy index of the questionnaire items to determine whether all the items and the number of items related to each dimension of the instrument are sufficient to measure infertile women’s quality of life [27]. The quantitative content validity of the QOL-QIW was investigated on the basis of the opinions of 10 experts (faculty members of the Nursing and Midwifery Faculty at Shahid Beheshti University of Medical Sciences) regarding relevance, simplicity, clarity, and necessity. The opinions were expressed using the Likert scale provided for each question, and the content validity ratio (CVR) was calculated.

A. CVR: To ensure the selection of the most important and correct contents (the necessity of the items), the narrative ratio (Lawshe, 1975) was calculated for each question. The experts signified their opinions by assigning each item scores of 1 to 3, which correspond to “not necessary,” “useful but not essential,” and “essential,” respectively. The scores were then calculated using this formula: the number of experts who provided “essential” scores minus half of the total number of raters divided by half of the total number of raters. That is,
  $$ CVR=\frac{N_e-\frac{N}{2}}{\frac{N}{2}} $$

The resultant value was compared with the values in Lawshe’s table. If the former exceeded the latter, the item to which this value was attached was considered essential for inclusion in the instrument, with a *P* = 0.05 regarded as indicative of statistical significance. The accepted value was determined on the basis of Lawshe’s table and the number of experts [28]. The opinions of the 10 experts were referred to in evaluating the CVR, with 0.62 regarded as acceptable.

B. Content validity index (CVI): This index was calculated in accordance with Waltz and Bausell’s (1983) criteria to ensure that the items of the instrument are excellently designed to measure content. The expert evaluation was focused on relevance, clarity, and simplicity and was expressed using a four-point Likert scale. Relevance pertains to whether a statement is irrelevant, requires modifications, is relevant but needs revision, or is completely relevant and appropriate (scores of 1 to 4, respectively). Simplicity refers to whether a statement is complicated, needs modifications, is simple but needs revision, or is completely simple and fluent (scores of 1 to 4, respectively). Clarity denotes whether a statement is vague, needs modifications, is clear but needs revision, or is completely clear and sensible (scores of 1 to 4, respectively).

The CVI score of each statement was calculated by dividing the number of experts agreeing with each statement using scores 3 and 4 on the Likert scale by the total number of experts. On the basis of this index, an entire statement was initially measured in terms of relevance, after which its acceptability was determined according to the following criteria [29, 30]:

- A CVI score over 0.79 is regarded as adequate.
- A CVI score of 0.7 to 0.79 is considered questionable and calls for revision and modification.
- A CVI score of 0.7 denotes unacceptability, and an item must be removed.

### Correction of the CVI formula to Verify the probability of chance agreement

The most important issue that should be addressed along with the CVI is the possibility that it matches chance agreement. Polit et al. (2007) proposed the modified Kappa statistics of K* and the CVI for this purpose. To calculate K*, the probability of chance agreement should first be computed through the following formula used for binominal random variables:

$$ {P}_C=\left[\frac{N!}{A!\left(N-A\right)!}\right]\times {0.5}^N $$

in which N indicates the number of raters, and A represents the number of agreements regarding relevance. Then, K* is calculated using the ratio of agreement on relevance or the individual CVI and the probability of chance agreement.

$$ {K}^{\ast }=\frac{I- CVI-{P}_C}{1-{P}_C} $$

K* is evaluated as excellent (K > 0.74), good (K = 0.60–0.74), or relatively good (K = 0.40–0.59).

### Qualitative evaluation of face validity

Face-to-face interviews with 10 infertile women were conducted to qualitatively evaluate the face validity of the QOL- QIW. Specifically, the evaluation was carried out with regard to the following:

A. Level of difficulty: Statements or words whose meaning is difficult for the participants to understand were identified.
B. Examination of relevance: The transferability and optimal relationship of the statements with the main objective of the scale and the dimensions of the questionnaire were examined.
C. Examination of ambiguity: The misconceptions of the statements or the semantic inadequacy of the words was scrutinized.

### Quantitative assessment of face validity

Next, the influence coefficient of the items was used to reduce the number of statements, remove inadequate ones, and determine the importance of each statement. First, a five-point Likert scale was incorporated into each item: 5 = *extremely important*, 4 = *important*, 3 = *moderately important*, 2 = *unimportant*, and 1 = *extremely unimportant*. The instrument was then distributed to 10 other infertile women to determine its quantitative face validity. The following formula was used in the calculations after the instrument was completed by the target group:

$$ \mathrm{Impact}\ \mathrm{score}=\mathrm{frequency}\ \left(\%\right)\times \mathrm{importance} $$

where *frequency* denotes the percentage of participants rating an item as 4 and 5, and *importance* refers to the mean score of significance based on the above-mentioned Likert scale. If the index was equal to or greater than 1.5, the item corresponding to this index was considered adequate for subsequent analyses and was retained [31].

### Construct validity

Exploratory factor analysis (EFA) was performed during a cross-sectional study to examine the latent constructs of the QOL- QIW. The sample size was five to 10 infertile women for each item of the questionnaire. Sampling adequacy was examined using the Kaiser–Meyer–Olkin (KMO) test, with values greater than 0.8 deemed acceptable. Bartlett’s test of sphericity was performed to ensure the adequacy of the data to be subjected to the EFA. The main factors were extracted in the first step of the analysis using rotation proportional to the data as well as the selection of the number of factors on the basis of eigenvalues greater than 1 and the scree plot. A minimum factor loading of 0.4 was considered necessary to warrant retaining a statement [32].

### Assessment of feasibility

The feasibility of the instrument was assessed to examine the association of the participant-related individual variables with the main research variable. Pearson’s correlation was used to determine whether the quantitative variables were normally distributed, while Spearman’s correlation was employed to identify whether the quantitative and qualitative ordinal variables were abnormally distributed. A correlation coefficient greater that 0.7 indicates high feasibility [27].

An instrument’s degree of ease indicates the extent to which a final instrument is convenient for respondents to complete (i.e., speed and credibility). The following formulas were used to calculate the degree of ease of the QOL- QIW.

$$ \mathrm{Percentage}\ \mathrm{of}\ \mathrm{items}\ \mathrm{answered}=\left(\mathrm{items}\ \mathrm{answered}\div \mathrm{total}\ \mathrm{items}\right)\times 100 $$

$$ \mathrm{Percentage}\ \mathrm{of}\ \mathrm{unanswered}\ \mathrm{items}=100\%-\mathrm{percentage}\ \mathrm{of}\ \mathrm{items}\ \mathrm{answered} $$

Here, values ranging from 30 to 70% were considered adequate [27].

### Ceiling and floor effects

Ceiling and floor effects are one of the indicators of an instrument’s interpretability. The ceiling effect occurs when most respondents select answers at the higher end of a scale, whereas the floor effect occurs when most participants choose responses at the lower end of the scale. To improve these items, a researcher can rewrite questionnaire statements and increase their difficulty by expressing them as “strongly positive” and “strongly negative” expressions [33]. This index should be less than 20% to cover all criteria and show changes over time [34]. The ceiling and floor effects of the total score of the questionnaire and the scores of all sub-scales were calculated as percentages to evaluate scale resolution and response distribution.

### Evaluation of internal consistency with Cronbach’s alpha coefficient

The Cronbach’s alpha coefficient was calculated after the instrument was developed and its construct validity was examined. A coefficient above 0.7 was deemed acceptable [35].

### Stability

Stability pertains to the acquisition of the same scores by a group of people at two different time phases. The important point in this method is the time interval between two tests, which can vary from 2 weeks to a month [35]. Accordingly, 20 eligible individuals completed the questionnaires twice in a 14-day interval, after which the intra-class correlation coefficient (ICC) was computed. A reliability coefficient greater than 0.7 was regarded as acceptable [32].

### Standard error of measurement

The standard error of measurement (SEM) is the most common method for ascertaining absolute reliability. An individual’s observed score is the score obtained from the test, and because the error is supposed to occur, such a score may be high or low. This means that if an individual passes the same test at another time, his/her score is likely to differ. A high reliability leads to a low difference between scores, whereas a low reliability results in a considerable difference in scores. The calculation of the SEM yields a confidence interval used to estimate the range of scores along which an individual’s actual score is located. The following formula was used for this purpose [31]:

$$ \mathrm{SEM}=\mathrm{SD}\times \surd 1-\mathrm{ICC} $$

### Minimal detectable change (MDC)

One of the common indicators for calculating the measurement error is the MDC, which is expressed in identical units as the measurement of results and is considered a component of absolute reliability. It is estimated through the SEM and defined as the smallest actual change (not the change in the measurement error). It was calculated using the equation below [27]:

$$ \mathrm{SEM}=\surd 2\times \mathrm{MDC}=\mathrm{Z}\ \mathrm{score} $$

### Sample size

Sample size was determined on the basis of the number of extracted items in the first stage of the study. It is sometimes suggested that the minimum sample size required for factor analysis is five to 10 samples for each item of a questionnaire [36].

### Research setting

The questionnaires were completed in an educational hospital affiliated with the Mazandaran University of Medical Sciences and a private center for infertility in Sari (north of Iran).

### Sampling method

In the quantitative stage of the research, convenience sampling was used to select and recruit the participants. The inclusion criteria are similar to the qualitative section.

### Data analysis

Data were entered into the Statistical Package for the Social Sciences (version 22) for analysis. A chi-square test, t-test, KMO test, and Bartlett’s test of sphericity were conducted. The ICC and Cronbach’s alpha were determined, and an EFA was carried out.

## Discussion

Infertility has numerous outcomes at the individual, family, and social levels. At the individual level, problems such as stress, anxiety, depression, sexual problems, and so on can lead to psychological imbalance, separation between couples and, ultimately, divorce; these outcomes, in turn, have strong negative effects on women’s quality of life [11]. In the culture of developing societies, patriarchal beliefs about the survival of a generation, the lack of social and economic support, low chances of remarriage for infertile women on the one hand and condemnation as to independent living on the other are some of the factors that cause discomfort and impose multiple pressures on women [37]. Quality of life is a health indicator that covers a combination of an individual’s knowledge regarding different aspects of life and performance in respect of human, work, and social relations. It is therefore essential for the optimal continuation of life and well-being. This concept is strongly influenced by demographic, social, economic, cultural factors as well as health- and disease-related variables [38], and it is a powerful force in directing, maintaining, and promoting the welfare of different societies and cultures [39].

Because childbearing and parenting have traditionally been the most prominent roles taken on by women, infertility is conventionally viewed as a female issue. As previously stated, infertile men and women differ in that the latter are more vulnerable to the condition, and their suffering is more severe and profound. Additionally, the social structures that cause this suffering and its consequences on quality of life among women differ from those occurring among men. Therefore, women can be expected to face psychological and social problems that diverge from those encountered by men. Despite these key differences, however, quality of life instruments—whether general or specific—neither particularly address the status of infertile women on the basis of female-related factors nor involve the use of new methods for measuring infertile women’s quality of life. Novel approaches can effectively facilitate the development and psychometric testing of reliable and valid instruments. These scales are paramount in the assessment of infertility-associated situations, enabling the use of correct information in planning and policymaking. The aforementioned deficiencies prompted the present study to qualitatively investigate the concept of quality of life as well as develop and psychometrically evaluate a reliable and valid instrument through an inductive and deductive approach combined with new measurement methods.

One of the limitations of this study is that the women did not fully express their feelings. Researchers should rectify this impediment by gaining the trust of respondents and establishing proper mutual communication. The strengths of the research are its sequential exploratory nature, its exploration of female-related factors of infertility, and its consideration of maximum variety in the sample.

## Data Availability

The datasets used and/or analyzed during the current study are available from the corresponding author on reasonable request.

## Abbreviations

WHO: World Health Organization
SF36: 36-Item Short Form Health Survey
WHOQOL: WHO Quality of Life Questionnaire
QOL-QIW: Quality of Life Questionnaire for Infertile Women
CVR: Content Validity Ratio
CVI: Content validity index
EFA: Exploratory Factor Analysis
KMO: Kaiser–Meyer–Olkin
SEM: The Standard Error of Measurement
ICC: The Intra-Class Correlation Coefficient
MDC: Minimal Detectable Change
